# On a New Operation for Obtaining Union of an Ununited Fracture, with Remarks on Its Application in Certain Cases of Recent Fracture

**Published:** 1864-11

**Authors:** E. R. Bickersteth

**Affiliations:** Surgeon to the Liverpool Royal Infirmary.


*On a New Operation for obtaining union of an Ununited Fracture, with Remarks on its Application in Certain Cases of Recent Fracture.* By E. R. BICKERSTETH, F. R. C. S., Surgeon to the Liverpool Royal Infirmary.

In bringing this subject before the attention of the Society, the author proposed to mention some cases that had occured in his practice to show the successive steps by which he arrived at the process in question. He had frequently tried, in vain, friction, accupuncture, and subcutaneous division; and though resection of the ends of the bone had been successful in some instances, it was a proceeding involving a considerable risk to life. Dieffenbach's method had proved to be more successful; but this operation, though conducive to the formation of new bone, in no way provided for what was of paramount importance—viz: absolute immobility of the opposing fragments. The large external wound and injury done to the soft parts in introducing the ivory pegs were also objections to this operation. Recognizing the happy influence of Dieffenbach's plan of exciting ossific deposit, and, at the same time feeling the importance of keeping the ends of the bone in a condition of absolute immobility, the author was induced to try a modification of the operation; and in the case of a man admitted under his care at the Liverpool Royal Infirmary with an ununited fracture of the radius, he drilled a hole through the ends of both fragments, and, passing a stout wire through it secured the bone in perfect apposition. Union took place in seven or eight weeks, but on endeavoring to remove the wire, so much traction was necessary that it caused the fracture again to be ununited. The difficulty of removing the wire induced the author to think of some other plan not open to this objection; and in the case of a man with an ununited fracture of the thigh, by means of a common Archimedean drill, he bored two holes in such directions that each passed obliquely through both ends of the fractured bone, and into each introduced a steel rod with a screw at the end. To do this it was necessary to make an incision three inches in length. Much constitutional disturbance followed, the wound suppurating freely. In ten weeks the splints were removed, but no union had taken place. The limb was then confined in gum-and-chalk bandages. Symptoms of pleuro-pneumonia came on, and he gradually sank. A post mortem examination showed tubercular deposit in the ends of the bone and other parts of the body. There was no attempt at repair at the seat of fracture, except where the drills had pierced the bone, and here there was a deposit of new bone. This proceeding showed that it was quite feasible to fix the bone in the manner described, without exciting too much inflammatory action; and also that the steel rods caused the formation of new bone.

The next case was a fracture of the lower maxilla, where the bones had united in such a position as to render the patient a most unsightly object. As the incision that would be necessary in this instance, for the purpose both of putting the bone into proper position and removing the deformity of the soft parts, would not allow the use of external splints or supports, and as it was found impracticable to effect this object by fixing the teeth by an appliance within the mouth, it was absolutely necessary that some means should be devised by which the divided portions of the jaw could be securely fixed; and it occurred to the author that pegs or nails would answer the purpose, especially as he had already observed their presence caused so little inconvenience. Accordingly, at the operation, the plan just mentioned was carried out, and the apposition of the fractured portions was secured by means of two round-headed nails. They most effectually answered their purpose, and no external splint or bandage was required. The case did well, no undue action being set up. On the twenty-second day after the operation, one of the nails came away. The patient left the Infirmary perfectly well, the jaw being firmly united in its proper position, and the deformity of the soft parts removed. One of the nails still remained in; and the last account states that its presence caused no inconvenience.

The third case recorded was one that presented many points in common with the one just narrated. No external incision was made, and ordinary drill heads were substituted for nails. The result was everything that the author could have wished.

These cases show how readily and with what good effect fractured bones may be fastened together. Surgeons have ever recognized the use of sutures with regard to the soft parts. Why should we not, in cases of difficulty arising from an inability to keep the surfaces in proper apposition, adopt the same plan with the bones? Might not this process be applicable in some cases where division of the tendo-Achillis is required, or where such an operation as sawing off the ends of the bone is indicated? From a consideration of the cases narrated, Mr. Bickersteth proposed to treat an ununited fracture by passing one or more drills through the broken ends of the bone in such a manner as to secure their perfect immobility, and without making any external wound beyond that caused by the entrance of the drill. The limb should then be secured by properly adjusted splints, and kept at perfect rest. After two or three weeks the drills may be removed, and water-dressing applied to the punctures. For several weeks after, it would, of course, be desirable to continue the use of the splints. In conclusion, the author begged to place upon record three cases of ununited fracture recently treated by his friend, Mr. Fletcher, on the plan that he (Mr. Bickersteth) had suggested, and in each the result had been most satisfactory.

Mr. Fergusson said that he scarcely remembered to have heard a paper of greater surgical interest than the one just read. It had the merit of bringing out much that was going on in the modern practice of surgery, and thought the paper would lead to greater improvements in practice. Here was further proof, he continued, of the advantage of wire and metal in instances in which in former times we were loth to use such materials. He had had the impression that ivory pegs, being softer, would be less likely to do harm; but now the author had shown that metal might be safely used. And it had been shown, too, that the commoner metals were as useful as the rarer; that iron wire was as useful as silver wire. In the same way, cauterization by an iron instrument was just as useful as by a gold or silver one. Dieffenbach had used the pegs of ivory to create irritation only, but the author had gone further, and fastened the bones together by pegs of iron. The author had shown that much might be done in desperate cases of ununited fracture. From hearing the cases related by the author, he should consider that the plan was safe, and that it ought to command attention. Mr. Fergusson said that he once saw Mr. Abernethy attempt to pass a seton between the ends of an ununited fracture. Failing to do so, he left a probe sticking in the wound between the ends of the bones. The result was good.

Mr. Holthouse thought the interest of the author's plan was not only in fastening the bone together, but in doing it subcutaneously. This was a great merit, and no doubt would lead to the adoption of the plan and to beneficial results in practice. Mr. Holthouse then gave the particulars of a case of ununited fracture of the humerus under his care at the Westminster Hospital, in which he had adopted the novel proceeding of inserting the sharpened end of the lower fragment into the cancellous structure of the upper, thus imitating an impacted fracture. This plan, however, did not succeed. The case was, however, a complicated one, there being anchylosis of the elbow.

Mr. Holmes Coote rose to correct what he believed was a very general and erroneous impression as to the views of Dieffenbach. This surgeon used to cut down to the ends of the bones and pass in ivory pegs, with the hope of creating irritation; but, if he could do it easily, he used also to fasten the ends of the bone together. Mr. Coote thought that three classes of cases ought to be distinguished; first, those in which there was union in good time; second, those in which union was simply retarded; and third, those in which union could not be obtained, as the ends of the bones were in a state of fatty degeneration. In the third class no good results could be hoped for.

Mr. Curling regarded the author's plan as ingenious, as a happy application of the modern treatment with metallic substances and as calculated to be of great service, not only in ununited fractures, but also in compound and in complicated simple fractures, when it was difficult to keep the parts in position. He was struck with the remark in the paper that the author had met with numerous cases of ununited fracture. Though Mr. Curling had been long attached to a large hospital where a great many fractures were admitted, he had seen only a few cases of ununited fracture, and all of them had been brought to the hospital in that condition, most of them being the result of accident which had occurred at sea. The author, being surgeon to the principal hospital in the chief seaport city in this country, probably met with an unusual number of such cases. Mr. Curling had seen, however, many cases of delayed union, and mentioned two remarkable instances of the kind, one a double fracture of the thigh, which happened at sea seven months before, and might have been considered an ununited fracture, but which got firm in a plaster-of-Paris case. He called attention to a constitutional indisposition to ossific union, independent of the general health, which was manifested in young people as well as in adults, and gave an instance in point of long delayed union in a fracture of the tibia in a girl.

Mr. Barwell, while agreeing with what had fallen from Mr. Holmes Coote concerning the degenerated condition of bones, considered that such condition was the result, more frequently than the cause, of non-union. It was a law of animal nature that any organ losing its function should degenerate. Thus when a bone lost its power of support, the surrounding muscular pressure and other conditions of its healthy life, it will surely degenerate; but until that degeneration had reached a high point it might still be restored. A remarkable case had occurred to him lately, which would also show that in certain instances the admirable plan proposed by Mr. Bickersteth would be unavailing—as in cases where the non-union was produced by a large quantity of soft parts intruding between the fractured ends. The case alluded to was as follows: About eighteen months ago a man broke his arm about two inches and a half above the elbow. He was admitted into the Charing Cross Hospital. The fracture united well, and the man was discharged cured. The same night, however, he got very drunk, and broke his arm again, but took no notice of the circumstance, continuing drunk for about a fortnight. Two months ago he again entered the hospital with a broken arm. As Mr Barwell had been for some time taking the duty of his colleague, Mr. Canton, this case came, about a fortnight ago, under his observation, and he determined to operate. The upper and longer fragment was on the outer side, its lower end overlapping the head of the radius. The inner end of the lower fragment was half-way up the inner side of the arm. The movement to and fro of this portion was very considerable; but there was always a wide interval between the two bones, which was occupied constantly by the anterior brachial muscle, sometimes also by part of the biceps, and in certain positions it seemed as though the artery and nerve also got between the fragments. The man having been placed under the influence of chloroform, Mr. Barwell made an incision two inches long over the outer fragment, and, turning out its end, sawed out a wedge-shaped piece, so as to leave an angular gap or notch in the end of the bone. The inner fragment lay so far away from the wound, and in such close proximity to the artery and nerve, that the greatest care was required in getting its end to protrude at the wound. This, however, was accomplished without any untoward accident, and the end was cut into a wedge shape, so as to fit with some degree of accuracy the interval in the upper fragment; traction was made upon the arm, and the two portions fitted together, and with the aid of a splint they retained perfect apposition. A singular condition of bone revealed itself during the operation—namely, that the periosteum on the upper fragment was loose, and could be slipped up off the bone as a man might turn up his shirt-sleeves. This tissue was carefully replaced, yet, on account of this condition of bone, he (Mr. Barwell) could not but look to the issue of the case with some anxiety. The man had as yet (ten days afterwards) no bad symptoms.

Mr. C. H. Moore thought the plan suggested would be valuable in some cases; but there would be some risk to life by tetanus and pyæmia.

Mr. Hilton said that, as a surgeon to a large hospital, he must add to the commendations of the other speakers his opinion that the paper was one of great interest, and that the plan was likely to be very useful when ordinary treatment had failed. He (Mr. Hilton) understood Mr. Holmes Coote to say that when there was fatty degeneration of the bone the ends never would or could unite; yet the author had related a case of cure by his plan, although at the operation the bone was so soft that a knife was passed through it.

